# Survival after in-hospital cardiopulmonary resuscitation in a major referral center

**DOI:** 10.4103/1658-354X.65131

**Published:** 2010

**Authors:** Masoud Saghafinia, Mohammad Hosein Kalatar Motamedi, Mohammad Piryaie, Hasan Rafati, Abdollah Saghafi, Alireza Jalali, Seyed Jalal Madani, Reza Bakhshi Kolahdehi

**Affiliations:** *Trauma Research Center, Baqiyatallah University of Medical Sciences, Tehran, Iran*; 1 *Department of Anesthesiology, Baqiyatallah University of Medical Sciences, Tehran, Iran*

**Keywords:** *Cardiopulmonary resuscitation*, *cardiopulmonary arrest*, *survival*

## Abstract

**Aim:**

This study was undertaken to assess the demographics, clinical parameters and outcomes of patients undergoing cardiopulmonary resuscitation (CPR), by the code blue team at our center to compare with other centers.

**Materials and Methods:**

Data were collected retrospectively from all adult patients who underwent CPR at our hospital from 2007 to 2008. CPR was performed on 290 patients and it was given 313 times. Clinical outcomes of interest were survival at the end of CPR and survival at discharge from the hospital. Factors associated with survival were evaluated via binomial and chi square-tests.

**Results:**

Of the 290 patients included, 95 patients (30.4%) had successful CPR. However, only 35 patients (12%) were alive at discharge. The majority requiring CPR were above 60 years of age (61.7%). Males required CPR more than females. There were 125 women (43.1%) and 165 males (56.9%) aged 3 to 78 (average 59.6) years. Majority (179) of the cases (61.7%) were above 60 years of age. Regarding the various wards, 54 cases (17.3%) were in the internal medicine ward, 63 cases (20.1%) in the surgery ward, 1 case (0.3%) in the clinic, 11 cases (3.5%) in the paraclinic, 116 cases (37.1%) in the emergency (ER), 55 cases (17.5%) in the Intensive Care Unit (ICU) and Coronary Care Unit (CCU), and 13 cases (4.2%) were in other wards. Cardiac massage was done in 133 cases (42.5%), defibrillation only via electroshock 3 cases (1%), and both were used in177 cases (56.5%). The ER had the most cases of CPR. Both cardiac massage and electroshock defibrillation were needed in most cases.

**Conclusion:**

In-hospital CPR for cardiopulmonary arrest was associated with 30.4% success at our center at the end of CPR but only 12% were alive at discharge. Duration of CPR >10 minutes was predictive of significantly decreased survival to discharge.

## INTRODUCTION

Cardiopulmonary resuscitation (CPR) has been widely practiced since the clinical utilization of closed chest massage was first reported in 1960.[1] Initial survival after CPR may exceed 50%, but hospital discharge rates are much lower.[2] Studies from the 1990s have noted hospital CPR discharge rates of 13–14%,[3,4] and a more recent Canadian study reported similar findings.[5] Using the data from 14,720 in-hospital cardiac arrests in the National Registry of Cardiopulmonary Resuscitation (NRCPR), Peberdy *et al*.[6] have reported overall survival to hospital discharge of 17%. A 17% survival rate to discharge was also reported by Tunstall-Pedoe *et al*.[7] who included arrests with onset outside the hospital. Recently, Nadkarni *et al*.[8] analyzed several years of NRCPR data to compare the survival outcomes of children and adults after cardiac arrest associated with different arrest mechanisms. Using survival to discharge as the primary outcome measure, the latter author found a rate of survival of 18% for adults after pulseless cardiac arrest. Matot *et al*.[9] also prospectively studied the effect of arrest time with hospital discharge as the primary outcome measure, and their finding is that survival to discharge was poorer after night shift CPR than after combined morning and evening shifts. Cardiac-respiratory arrest is a foremost problem in many medical centers worldwide and CPR is part of the responsibility of the code blue anesthesia team and anesthesia department. This study was undertaken to assess the demographics, clinical parameters and outcomes of patients undergoing CPR by the code blue team at our center.

## MATERIALS AND METHODS

Data relevant to CPR outcome in 313 hospitalized patients coded blue (code 99) at our hospital were gathered and analyzed. Regarding the various wards, the internal medicine ward, surgery ward, clinic, paraclinic, emergency (ER), ICU, CCU and other wards were assessed. CPR data documented in the CPR sheet by the hospital supervisor in 290 patients were gathered. Data consisted of the patient‘s name, age, sex, ward, the time code was called, initiation, duration and repetition of CPR, devices and drugs used, shift (night or day), holiday or working day, and outcome to discharge. An anesthesiologist, cardiologist, anesthesiologist technician, nurse and supervisor were present in all cases of CPR. The CPR report and personal data registered were verified by the hospital supervisor and nurses‘ office and analyzed via SPSS software using binomial and chi-square tests.

## RESULTS

Data were collected retrospectively from all patients who underwent CPR at our hospital from 2007 to 2008. CPR was performed on 290 patients; 125 were females (43.1%) and 165 were males (56.9%). CPR was done 313 times. The patients were aged 3 to 78 (average 59.6) years. There were 33 patients under 30 years (11.4%); 78 cases were aged 30–60 years (26.9%) and 179 cases (61.7%) were above 60 years of age. The success rate was greater in males OR = 1.062, CI=95% (0.65, 1.74); 54 cases (17.3%) were in the internal medicine ward, 63 cases (20.1%) in the surgery ward, 1 case (0.3%) in the clinic, 11 cases (3.5%) in the paraclinic, 116 cases in the emergency room (ER) (37%), 55 cases (17.6%) in the ICU and CCU, and 13 cases (4.2%) were in other wards [Table 1]. Clinical outcomes of interest were survival at the end of CPR and survival at discharge from the hospital. Factors associated with survival were evaluated using t-test and chi-square test. Multiple CPR was required in 23 patients [Table 2] (7.9%). Of the 290 patients included, 95 patients (30.4%) were alive after CPR and 35 (12%) were alive at discharge. The ER had the most cases of CPR. Two hundred thirty-four cases were resuscitated from 7 a.m. to 7 p.m. and 56 underwent CPR between 7 p.m. and 7 a.m. CPR in day shifts (7 a.m.–7 p.m.) was significantly higher than that during night shifts OR = 1.47, CI = 95% (0.8, 2.7), indicative of the fact that more invasive procedures are done in day shifts. Additionally, 53 cases (18.3%) were resuscitated on holidays and 237 (81.7%) on working days. CPR on working days was significantly higher than on holidays (*P* < 0.0001).

**Table 1:** Immediate CPR results in various wards

Wards	Successful (%)	Unsuccessful (%)
Internal medicine	19 (35.2)	35 (64.8)
Surgery	15 (23.8)	48 (76.2)
Clinic	0	1 (100)
Paraclinic	4 (36.4)	7 (63.6)
EMS	34 (29.3)	82 (70.7)
ICU and CCU	18 (32.7)	37 (67.3)
Others	5 (38.5)	8 (61.5)
Total	95 (30.4)	218 (69.6)

**Table 2:** Repetition of CPR and outcomes

Repetitions	Number of patients	Number of patients alive at discharge	Number died (%)
x2	20	2	18(90)
x3	3	0	3(100)
Total	23	2	21(91)

The essential cause for CPR was: respiratory disease 63 cases (20.1%); cardiac disease 60 cases (19.2%); oncology 51 cases (16.3%); liver and gastrointestinal (GI) disease 42 cases (13.4%); neurologic disorders 37 cases (11.8%); infectious disease 28 cases (9%); hematologic problem 11 cases (3.5%); metabolic disease 8 cases (2.6%); anaphylaxis 1 case (0.3%) and trauma 12 cases (3.8%) [Table 3].

**Table 3:** Causes for calling-in the code blue team

Cause	Number	Percent
Cardiac arrest	36	11.5
Respiratory arrest	40	12.8
Both	237	75.7
Total	313	100

The duration of CPR was 10 minutes or less in 95 cases (30.4%), 10–20 minutes in 49 cases (15.7%), 20-30 minutes in 81 cases (25.9%) and 30-60 minutes in 88 cases (28%). Duration of CPR >10 minutes was predictive of significantly decreased survival to discharge (*P* > 0.0001).

Cardiac massage only (most cardiac arrests) was done in 133 (42.5%) patients, defibrillation via electroshock (i.e., ventricular fibrillation, ventricular tachycardia, etc.) in 3 (1%) patients and both in 177 (56.5%) patients.

Intubation was required in 124 cases (39.6%) and mechanically assisted ventilation with intubation in 189 cases (60.4%).

## DISCUSSION

This study was undertaken at a major referral hospital with 800 beds and all subspecialties and full array of diagnostic and treatment facilities. Over 100 operations (average) are performed daily at this center (including cardiac surgery). An average of 0.7–1 case of CPR is seen daily at the general wards. CPR is more common in males than females; 61.7% of patients are in their sixth decade. CPR in day shifts was more than night shifts possibly because the interventions and procedures that may induce cardiac arrest were done in day shifts. Overall survival to discharge after in-house CPR in the current study was similar to that of previous studies.[6,8,10]

The prevalence of ventricular fibrillation or ventricular tachycardia was 11.5% in the current study, ventricular tachycardia was 20.3% in the previous study and prevalence of asystole was 68.8%. In one study, asystole was 71% and pulseless activity (PEA) was 25%.[10] These findings are similar to those of Nadkarni *et al*.[8] who reported 23% prevalence of ventricular fibrillation or ventricular tachycardia, and asystole and PEA prevalence of 35% and 32%, respectively, in adults. Cardiac massage was done in 133 patients (42.5%), defibrillation via electroshock in 3 patients (1%) and both in 177 of our patients (56.5%). Discharge after initial pulseless rhythm of ventricular fibrillation (28% (or ventricular tachycardia (13%) in a previous study[10] was similar to previous findings of Cooper *et al*.[11](survival of 33 and 31% with these initial rhythms, respectively). An initial observed rhythm of either ventricular fibrillation or tachycardia in adults has been reported to be favorable for survival to discharge compared to other rhythms.[9–18]

Multiple cardiac arrests have not been isolated as a predictor of poor outcome in some studies. Nadkarni studied only the first, or index, arrest.[8] Bialecki and Woodward found 18% survival of a second CPR and stated that a patient who has survived initial CPR should be considered for a second resuscitation if clinically warranted; but they did not consider multiple arrests as a factor influencing outcome. We saw a trend toward significant difference in survival to discharge between those with single arrest versus those with multiple arrests. In our study, 23 patients (7.4%) had more than one arrest, with only 14 (4.8%) surviving to discharge [Table 2]. Although duration of CPR was a significant factor in this study in predicting survival after cardiac arrest, the ability to dictate a prescribed maximum duration of CPR remains questionable especially with regard to medical ethics. Absolute accuracy of time documentation is difficult with standard methods.[17–19]. Facial and tracheal deformities are other factors that may also influence outcomes.[20]

**Figure 1:**
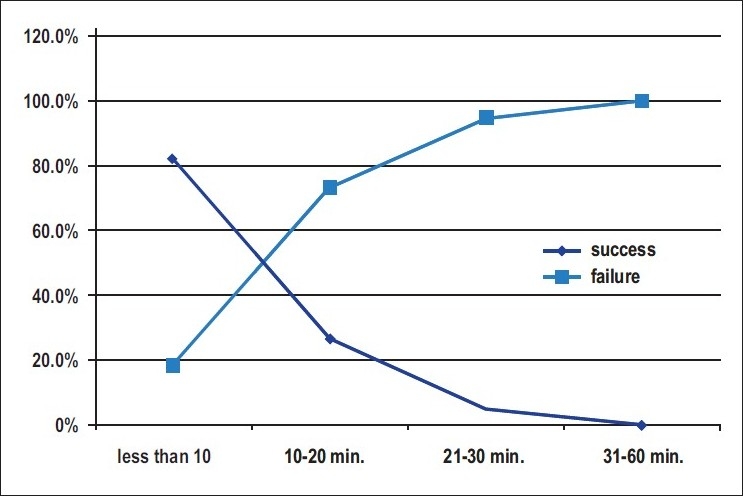
CPR success relevant to resuscitation time

A report from 115 studies showed a survival to discharge rate of 15.2% (USA 15%, Canada 16%, UK 17% other EU countries 14%).[18] Some studies have found that resuscitation longer than 15 minutes was associated with significantly decreased survival to discharge.[12] Duration of CPR of <10 minutes had a significant effect on survival to discharge in our study as calculated by multivariate analysis [Figure 1]. The average age in the previous study by Bialecki was 69 years, but the average age was (59.6 years) in our study. Our overall survival to discharge after CPR was 12%. Zoch *et al*.[12] reported an overall survival to discharge after CPR of 32%, which was substantially higher than the rate of 17% found by Peberdy *et al*.[6] These investigators speculated that increased use of “do not resuscitate” or “No Code” orders during the study period may have influenced the results. We do not use “do not resuscitate” order in our hospital.

## CONCLUSION

This study provides a retrospective report of survival after in-hospital pulseless cardiac arrest. Our data and findings were generally similar to the results of others studies in the current literature. It seems prudent to have a baseline of data and to seek ways to improve the outcome of in-hospital CPR.
